# Microtensile Bond Strength of CAD-CAM Restorative Dental Material Blocks to Resin Cement: An In Vitro Study

**DOI:** 10.3390/ma16134796

**Published:** 2023-07-03

**Authors:** Eva González-Angulo, Lucía Fernández-Estevan, Javier Casas-Terrón, Gisela Senent-Vicente, Carla Fons-Badal, Fernando García-Sala Bonmatí, Rubén Agustín-Panadero, Juan Luis Román-Rodríguez

**Affiliations:** Prosthodontic and Occlusion Unit, Department of Stomatology, Faculty of Medicine and Dentistry, Universitat de València, C/Gascó Oliag n° 1, 46010 Valencia, Spainjavier.casas@uv.es (J.C.-T.); gisela.senent@uv.es (G.S.-V.); carla.fons@uv.es (C.F.-B.); fernando.garcia-sala@uv.es (F.G.-S.B.);

**Keywords:** ceramic, sandblasted, dental materials, dental bonding, prosthodontics

## Abstract

Introduction: Today’s dentistry frequently employs bonded partial restorations, which are usually fabricated in ceramic materials. In the last decade, hybrid materials have emerged that attempt to combine the properties of composites and ceramics. Objectives: To evaluate in vitro, by means of a microtensile test, the bond strength between CAD-CAM restorative materials and the cement recommended by their manufacturer. Material and Method: From blocks of CAD-CAM restorative material bonded to composite blocks (Filtek 500^®^), beams with a bonding area of approximately 1 mm^2^ were made and divided into four groups: EMAX (IPS e.max CAD^®^ lithium disilicate), VE (VITA Enamic^®^ polymer-infiltrated ceramic matrix), LUA (Lava Ultimate^®^ nano-ceramic resin with sandblasting protocol) and LUS (Lava Ultimate^®^ nano-ceramic resin with silica coating protocol). In each group, perimeter (external) or central (internal) beams were differentiated according to the position in the block. The samples were tested on the LMT 100^®^ microtensile machine. Using optical microscopy, the fractures were categorized as adhesive or cohesive (of the restorative material or composite), and the data were analysed with parametric tests (ANOVA). Results: The LUS group had the highest results (42 ± 20 MPa), followed by the LUA group (38 ± 18 MPa). EMAX had a mean of 34 ± 16 MPa, and VE was the lowest in this study (30 ± 17 MPa). In all groups, the central beams performed better than the perimeter beams. Both EMAX and VE had the most adhesive fractures, while LUA and LUS had a predominance of cohesive fractures. Conclusions: Lava Ultimate^®^ nanoceramic resin with the silica coating protocol obtains the best bond strength values.

## 1. Introduction

Bonded partial restorations are currently a therapeutic alternative for treating cases of dental structure loss, regardless of their origin, avoiding the use of full veneer crowns that entail a 20–30% higher loss of structure [1,2]. Ceramics have been classically used to manufacture these restorations, as it provides adequate aesthetics but has a modulus of elasticity that differs significantly from that of dentin [3]. Composites, thanks to their modulus of elasticity [4], behave similarly to dentine, but they have lower mechanical strength and are more susceptible to abrasion [5]. Hybrid materials have been developed with the idea of combining the positive characteristics of ceramics and composites [6].

Currently, CAD-CAM (Computer-Aided Design—Computer-Assisted Manufacturing) materials can be differentiated [7] between glass-matrix ceramics, polycrystalline ceramics and resin-matrix ceramics, also labelled by some authors as hybrid materials [8,9,10]. Among the glass-matrix CAD-CAM ceramics, IPS e.max CAD^®^ (Ivoclar Vivadent, Schaan, Liechtenstein) made of lithium disilicate (LDS) stands out [11,12]. On the other hand, two groups of hybrid materials can be distinguished: nano-ceramic resins (NCR) and polymer-infiltrated ceramic network (PICN). NCR restorative materials contain silica nanomers (20 nm), zirconia nanomers (4 to 11 nm), nanocluster particles derived from the nanomers (0.6 to 10 μm), silane coupling agent, and resin matrix [13]. The NCR used in this study (Lava Ultimate^®^, 3M ESPE, St. Paul, MN, USA) is composed of 80% wt zirconia/silica nanoceramic particles embedded in a highly cross-linked resin matrix (20% wt) [14]. VITA Enamic^®^ (VITA Zahnfabrik, Bad Säckingen, Germany) is the first PICN to appear on the market. This material is composed of 86% wt of an inorganic feldspathic ceramic matrix, infiltrated by a monomer and subsequently polymerised. The organic polymeric part accounts for 14% wt and consists of UDMA (urethane dimethacrylate) and TEGDMA (triethynel glycol dimethacrylate) [15,16].

The increasing variety of materials with different compositions and physical properties leads to the development of new studies to better understand their behaviour and improve clinical procedures. This study analyses the bond strength between the restorative material and the cement.

The aim of this work was to analyse the microtensile bond strength of three materials indicated for indirect posterior restorations. The working hypothesis was that the ceramic material would have a higher bond strength to resin than the hybrid materials tested.

## 2. Material and Methods

A lithium disilicate (IPS e.max CAD^®^) and two hybrid materials were evaluated: an NCR (Lava Ultimate^®^) and a PICN (VITA Enamic^®^). Each of them was bonded to a composite block using the cement selected by the manufacturer, and four test groups were formed: EMAX (IPS e.max CAD^®^), which was used as the control group; VE (VITA Enamic^®^); LUA (Lava Ultimate^®^ with surface sandblasting); and LUS (Lava Ultimate^®^ with surface silica coating).

The composite blocks were manufactured with Filtek 500^®^ (3M ESPE) in 2 mm increments over a custom-made silicone mold (Elite^®^ HD+, Zhermack S.p.A, Badia Polesine, Italy) left by the restorative block. 

**Figure 1:**
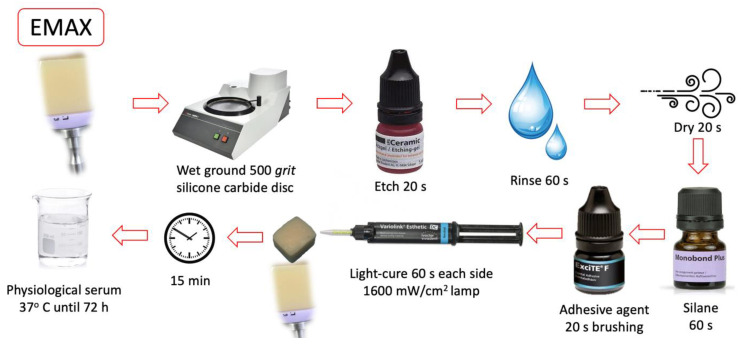
EMAX group cementing protocol.

**Figure 2:**
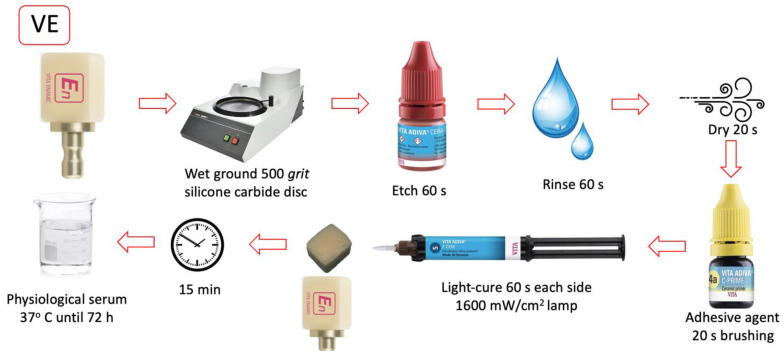
VE group cementing protocol.

**Figure 3:**
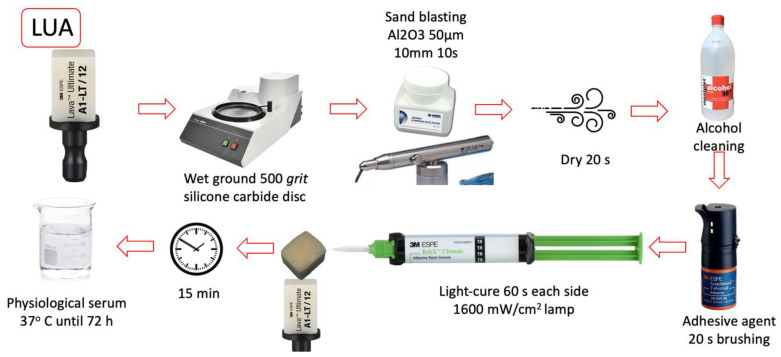
LUA group cementing protocol.

**Figure 4:**
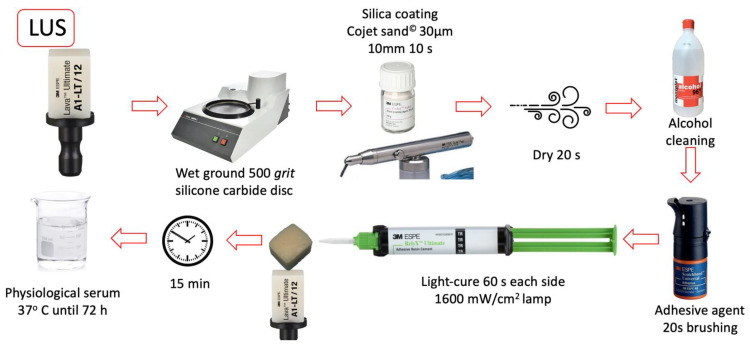
LUS group cementing protocol.

All bonding surfaces were polished with a 500-grit silicon carbide disc (Struers^®^ LaboPol-1. Struers ApS, Ballerup, Denmark). The bonding of the restorative material to the composite blocks was performed according to each manufacturer’s protocol. The materials used in the study and the listed steps followed in the cementation sequence are shown in Table 1. (Figure 1, Figure 2, Figure 3 and Figure 4).

**Figure 5:**
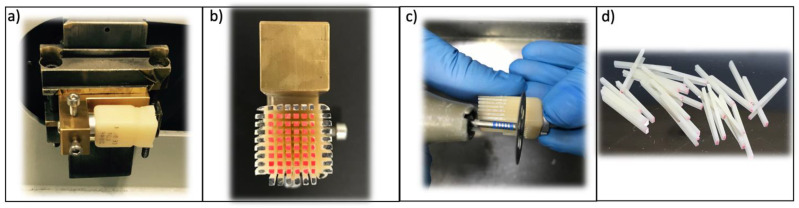
Manufacture of the test beams. (**a**) Cemented block in the cutting machine. (**b**) Cut-up block with marked beams: perimetral (black) and central (red). (**c**) Separation of the beams from the base of the block. (**d**) Group of beams.

The cemented blocks were kept for 72 h in saline solution at 37 °C and then sectioned with a cutting machine (Struers^®^ Accutom-10) to obtain beams with cross-sectional areas of 1 mm^2^. Each beam was numbered, calibrated, and identified as central or perimetral (Figure 5).

**Figure 6:**
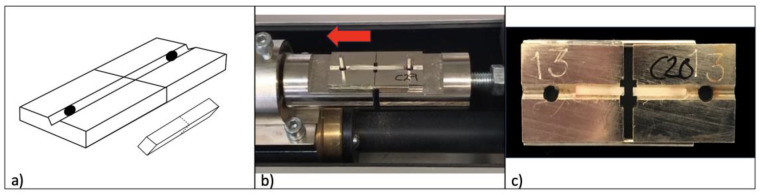
Microtensile test. (**a**) Schematic representation of a plate and a beam. (**b**) LMT100^®^ machine before test. (**c**) Broken beam.

The microtensile bond strength testing was carried out with an LMT100^®^ machine (LAM Technologies, Florence, Italy), with a crosshead speed of 0.5 mm/min until fracture occurred (Figure 6). The data obtained in the machine were expressed in N, and by relating them to the adhesion area of each beam, the results were obtained in MPa.

**Figure 7:**
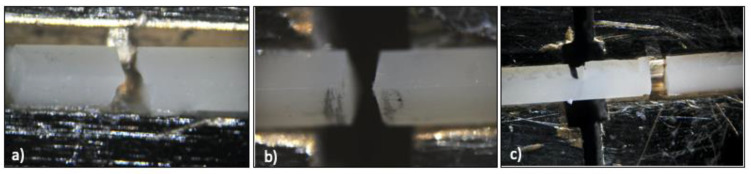
Fracture types: (**a**) material cohesive, (**b**) adhesive, (**c**) composite cohesive.

The fractured beams were observed under light microscopy (10×) (Nikon^®^ SMZ-10, Nikon, Tokyo, Japan), and the type of failure was classified as an adhesive (Figure 7b) or cohesive, from the restorative material (Figure 7a) or composite (Figure 7c).

All the data obtained were subjected to statistical analysis using SPSS Statistics software v22.0. The normality of the strength measures was tested using the Kolmogorov–Smirnov test and confirmed in all groups. In addition, the homogeneity of variances was verified using Levene’s test. A one-way general linear analysis of variance (ANOVA) model with group factor (protocol type) was developed, applying Bonferroni as a post-hoc test to compare the mean strength between groups. To assess the effect of beam position, the model was extended to a two-way ANOVA, evaluating the interaction effect between position and group and with the same type of comparisons (Bonferroni). The chi-squared test was used to measure the degree of association between fracture type and material group. On test F of the variance analysis model, with a confidence level of 95% and taking into account the size of the effect f = 0.25, the power achieved was 94.5%.

## 3. Results

The bond strength averages are shown in Table 2. The group with the best mean value was LUS (42 ± 20 MPa), followed by LUA (38 ± 18 MPa). The group with the lowest values was VE (30 ± 17 MPa), and EMAX had values of 34 ± 16 MPa. The LUS group showed notable differences with both VE and EMAX, and the LUA group only showed appreciable differences with the VE group. The numerical variety in the N of each group was due to the complexity of the sample preparation and the fracture of some beams during the preparation.

**Figure 8:**
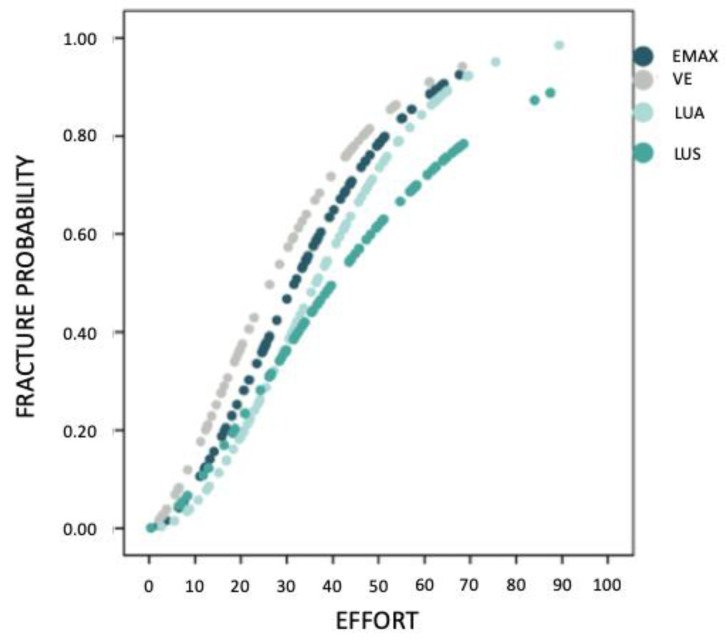
Fracture probability estimates.

After the Weibull calculation and analysis, the fracture probability of the different groups was estimated, and a graphical representation of the probability curve as a function of stress was made to allow comparison between the different materials (Figure 8). The LUS group is shown to be the best of all, as its curve is the rightmost. In addition, its slope is the smoothest of the four, with the highest Weibull modulus (m) (10.76), which indicates that the stress must be increased considerably to achieve a significant increase in fracture probability.

Table 3 shows the values obtained for the characteristic stress (σ_0_) of each of the materials and the Weibull modulus (m). EMAX is the one with the highest value in characteristic stress (202.05 MPa), which indicates that it is the strongest of all the materials studied.

**Figure 9:**
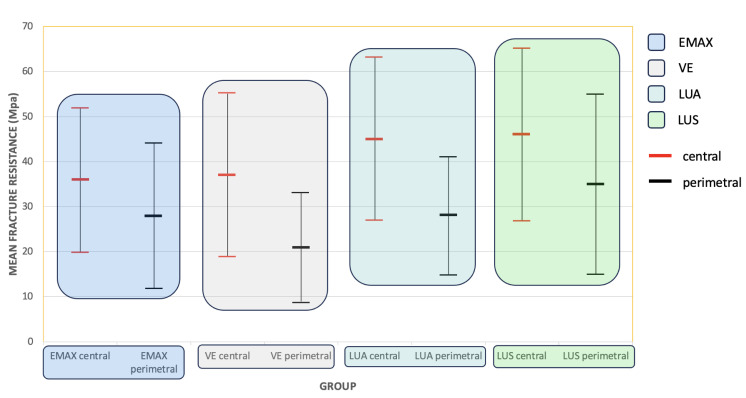
Estimated marginal means and standard deviation of bond strength (MPa).

When analysing the effect of beam position on bond strength, a two-way ANOVA model again showed that the group of material has an influence on the average strength, and that this influence is similar whether working with a perimeter or central beams. Furthermore, it showed that there is a position effect, as the central beams resist more than the perimeter beams (41 ± 18 MPa versus 28 ± 16 MPa). As Figure 9 shows, in any group, the test with central beams presents higher strength values than perimeter beams.

**Figure 10:**
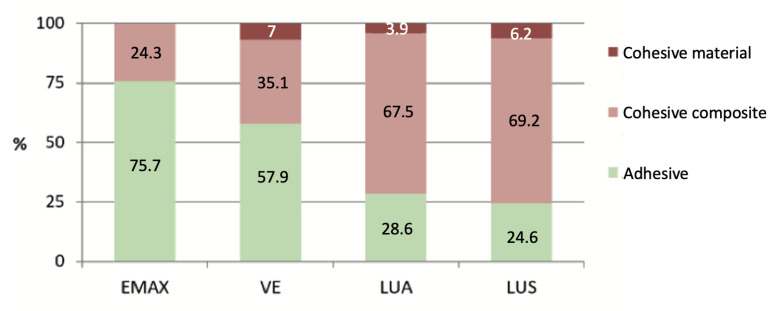
Fracture type according to its group.

When analysing the relationship between the type of fracture and the study group, as shown in Figure 10, in the two groups of Lava Ultimate^®^, there is a clear predominance of cohesive fractures, while in the ceramic and the hybrid material that most resemble it, VITA Enamic^®^, there is a majority of adhesive fractures, exceeding 75% in the EMAX group.

## 4. Discussion

This study was designed to evaluate the microtensile bond strength of two hybrid restorative materials (Vita Enamic^®^ and Lava Ultimate^®^) by comparing them with the adhesion of lithium disilicate (IPS e.max CAD^®^), whose bond strength has been extensively tested [3,16,17,18]. The chosen hybrid materials are the ones that have been on the market for the longest time, and there are sufficient scientific publications supporting their correct properties [19,20]. For all materials, the protocol recommended by the manufacturer was applied, which, a priori, will give the best results [21,22,23].

Analysing the results, VE achieves similar values to EMAX. Since the lithium disilicate ceramic bond to a resin cement has been shown in numerous in vitro and clinical studies to be strong, durable, and predictable [18,24], it can be inferred that the VE bond will have similar behaviour. Furthermore, the bond values achieved by LUS and LUA allow us to state that their NCR-cement bond will have these same characteristics or even better since their bonding values are superior. The results obtained in this test led to the partial rejection of the working hypothesis since the Lava Ultimate^®^ material, with any of the protocols, obtained better results than the ceramic material. An analysis of the scientific literature reveals a wide disparity in bonding protocols, which makes it difficult to compare bond strength results. Regarding the influence of different types of surface treatments, Frankenberger compared IPS e.max CAD^®^, VITA Enamic^®^, and Lava Ultimate^®^ using different bonding protocols for each of them [21]. The best values for each group were obtained using the protocols recommended by each manufacturer. The lithium disilicate ceramic achieved the highest bond strength value, with the nanoceramic resin having the lowest values, unlike those obtained in the present study. Elsaka compared the bond strength of Lava Ultimate^®^ and VITA Enamic^®^ bonded to a self-adhesive cement (Bifix SE, VOCO; Cuxhaven, Germany), applying different surface treatment protocols [25]. In that study, VITA Enamic^®^ showed a similar behaviour to the one found in the present study. However, Lava Ultimate^®^ obtained lower values, possibly due to the type of cement used. Colombo analysed the bond strength obtained by IPS e.max CAD^®^ and VITA Enamic^®^ by performing different etching protocols, varying the concentration and application time of hydrofluoric acid [26]. His results do not coincide with the manufacturer’s recommendations, as the best values in each of the groups were obtained with the use of 10% hydrofluoric acid for 20 s. Peumans carried out a study similar to ours with a different statistical model, and his results were the same: the etching and silane protocol is the one that shows the best results with IPS e.max^®^ CAD and VITA Enamic^®^ [27]. Bayazit tested VITA Enamic^®^ and Lava Ultimate^©^ with a different methodology, and his best results were achieved via area treatments different from those recommended by the manufacturer [28].

In the surface treatment of the nanoceramic resin, the manufacturer of Lava Ultimate^®^ recommends either sandblasting or silica coating of the resin. Therefore, in this research, two groups were created to assess whether there were differences between the two. The values obtained by silica coating were slightly higher, although the difference was not significant. Sandblasting is normally used to increase the bonding surface of the restoration and thus improve adhesion. Depending on the particle size chosen for sandblasting, the surface roughness will be different, and the larger the particle size used, the more irregular the surface [29]. In this study, the manufacturer’s recommendations were followed, the LUA group was sandblasted with 50 µm aluminium oxide particles, and the LUS group was treated with 30 µm silica-coated aluminium oxide particles. By using the latter type of particles, the silica remains incorporated into the outermost layer of the material [16] and enables better ceramic-cement chemical bonding due to the bond between the silica, the silane, and the resin cement [30]. This could be the reason for a slightly better result in the silica-coated group.

Adhesive fractures were predominant in both the EMAX and VE groups, while LUA and LUS had a majority of cohesive fractures. These results, together with the statistical analysis confirming that cohesive composite fractures were associated with significantly higher mean strength, lead to the conclusion that the bond strength of Lava Ultimate^®^ is even higher since if the weak element, the composite, did not fail in the experiment, even better values would be obtained. Only the hybrid materials had cohesive failures, while the IPS e.max CAD^®^ group, as expected, did not have any cohesive material failure since lithium disilicate is stronger than the hybrid restorative materials [31].

The differentiation of the study samples into perimetral or central, depending on where they were located within the beam preparation block, was done to analyse whether there was a difference in the behaviour of the outermost part of the restorations. In previous similar studies, it was found that there were two tendencies, either to discard the perimeter beams [25,26,27] or to include them without differentiating from those located in the central area [21,22,28]. The results obtained in this study showed that in all groups, the central beams obtained notably higher bond strength than the perimeter beams. The reasons for lower bond strength on the margins probably should be related to the specimen’s manufacturing process, like gaps around the margin blocks.

One of the limitations of this study is that only the bond strength between the cement and the restorative material has been analysed. It would be necessary to extend this test by incorporating the cement-tooth interface in the adhesion study and a cyclic fatigue test [32]. Due to the difficulty of extrapolating in vitro results to a clinical situation, in vivo studies would be necessary to analyse the behaviour of these new-generation materials.

## 5. Conclusions

With the limitations presented in this study, the following can be concluded:

1. All materials used with the bonding protocols recommended by their manufacturers and evaluated in this study achieve clinically adequate bond strengths.
2. Lava Ultimate^®^ is the material that, when used with its bonding protocol, achieves the best results in bond strength. Slightly higher values are obtained when silica coating rather than sandblasting.

In all groups, the central beams obtain better values than the perimeter beams.

## Figures and Tables

**Table 1:** Materials used in the study (BisGMA, Bisphenol A-glycidyl methacrylate; HEMA, polymacon; TEGDMA, triethylene glycol dimethacrylate).

Group	Material and Cementation Sequence	Type	Chemical Composition	Duration	Manufacturer’s Data	Lot Number
EMAX	1. IPS Ceramic etching gel	Ceramic acid etching	Hydrofluoric acid 4.9%	20 s	Ivoclar Vivadent	T76221
	2. Monobond Plus	Silane	Adhesive monomers 4%, Ethanol 96%	60 s	Ivoclar Vivadent	X43365
	3. Excite	Bonding agent	Phosphonic acid acrylate, dimethacrylates, hydroxyethyl methacrylate, highly dispersed silicon dioxide, ethanol, catalysts, stabilizers, and fluoride	20 s agitate	Ivoclar Vivadent	Z33289
	4. Variolink Esthetic DC Neutral	Dual resin cement	Barium glass filling, mixture of oxide 52.2%, dimethacrylate 22%, high dispersion silica, ytterbium trifluoride 25%, initiators and stabilizers 0.8%, pigments <0.1%	60 s each side	Ivoclar Vivadent	W95568
VE	1. VITA Adiva Cera-Etch	Ceramic acid etching	Hydrofluoric acid 5%	60 s	VITA Zahnfabrik	G32620
	2. VITA Adiva C-Prime	Silane	Metracrylsilane solution in ethanol	60 s	VITA Zahnfabrik	I18534
	3. VITA Adiva F-Cem	Dual resin cement	Mixture of bis-GMA-based resins, catalysts, stabilisers, pigments, and inorganic filler particles in a distribution of 0.05–1 μm	60 s each side	VITA Zahnfabrik	F72621
LUA	1. Rondoflex	Sandblasting powder	Aluminium oxide powder, particle size: 50 μm, pressure: 2.0 bars	15 s	KaVo	041025
	2. Scotchbond Universal	Universal bonding agent	HEMA 2-hydroxyethyl methacrylate; MDP 2-methyl-, 2-propenoic acid, reaction products with 1, 10-decanediol and phosphoric oxide (P_2_O_5_), (1-methylethylidene)bis [4,1-phenylenxy(2-hydroxy-3,1-propanediyl) bismethacrylate, Decamethylene dimethacrylate	20 s agitate	3M ESPE	4636140
	3. RelyX Ultimate	Dual resin cement	Base paste: silane-treated glass powder, 2-propenoic acid, 2-methyl, reaction products with 2-hydroxy-1,3-propanedyl dimethacrylate and phosphorus oxide, TEGDMA, silane-treated silica, oxide glass chemicals, sodium persulfate, tertbutyl peroxy-3.5,5-trimethylhexanoate, copper acetate monohydrate Catalyst paste: silane-treated glass powder, substituted dimethacrylate, 1.12-dodecane dimethacrylate, silane-treated silica, 1-benzyl-5-phentyl-barbic-acid, calcium salt, sodium p-toluenesulfinate, 2-propenic acid, 2-methyl-, di-2.1-ethanediyl ester, calcium hydroxide, titanium dioxide	60 s each side	3M ESPE	4751537
LUS	1. Cojet Sand	Silica coating powder	Silica-coated aluminium oxide powder, particle size: 30 μm, pressure: 2.0 bars	15 s	3M ESPE	3454446
	2. Scotchbond Universal	Universal bonding agent	HEMA 2-hydroxyethyl methacrylate; MDP 2-methyl-, 2-propenoic acid, reaction products with 1, 10-decanediol and phosphoric oxide (P_2_O_5_), (1-methylethylidene)bis [4.1-phenyleneiminoxy(2-hydroxy-3.1-propanediyl)] bis-methacrylate, Decamethylene dimethacrylate	20 s agitate	3M ESPE	4636140
	3. RelyX Ultimate	Dual resin cement	Base paste: silane-treated glass powder, 2-propenoic acid, 2-methyl, reaction products with 2-hydroxy-1.3-propanedyl dimethacrylate and phosphorus oxide, TEGDMA, silane-treated silica, oxide glass chemicals, sodium persulfate, tertbutyl peroxy-3.5,5-trimethylhexanoate, copper acetate monohydrate Catalyst paste: silane-treated glass powder, substituted dimethacrylate, 1.12-dodecane dimethacrylate, silane-treated silica, 1-benzyl-5-phentyl-barbic-acid, calcium salt, sodium p-toluenesulfinate, 2-propenic acid, 2-methyl-, di-2.1-ethanediyl ester, calcium hydroxide, titanium dioxide	60 s each side	3M ESPE	4751537

**Table 2:** Bond strength (MPa) as a function of group.

		GROUP		
	EMAX	VE	LUA	LUS
N	70	57	77	65
Mean	33.68	29.68	38.17	42.07
SD	16.27	17.26	18.36	19.67

**Table 3:** Fracture probability by group: characteristic stress values (MPa) and Weibull modulus (m).

		GROUP		
	EMAX	VE	LUA	LUS
Characteristic stress σ_0_	202.05	33.76	177.63	156.28
Weibull modulus m	7.59	1.48	8.64	10.76

## Data Availability

Information is available on request in accordance with any relevant restrictions (e.g., privacy or ethics).
